# Supplementary material for: Changes in the Phenotype and Metabolism of Peritoneal Macrophages in Mucin-2 Knockout Mice and Partial Restoration of Their Functions In Vitro After L-Fucose Treatment
Source: Int J Mol Sci. 2024 Dec 24;26(1):13. doi: 10.3390/ijms26010013 (PMC11719744; doi:10.3390/ijms26010013)
Supplement: Supplementary file 1 [file ijms-26-00013-s001.zip › fig captions.pdf]

Supplementary Figure S1 Macrophage markers expression in the colon and gating strategy of macrophages derived from C57BL/6 and Muc2−/− mice. (A). Percentage of F4/80+ macrophages in the colon of two mouse strains. (B). Percentage of CD80+ macrophages (M1-like type) and CD209+ macrophages (M2-like type) in the colon of Muc2−/− mice mice. (C). Gating strategy for F4/80+CD80+ (M1-like type) and F4/80+CD209+ (M2-like type) macrophages derived from C57BL/6 and Muc2−/− mice

Supplementary Figure S2. Ultrastructures of cells from the lamina propria of colons derived from and Muc2−/− mice mice. (A). Eosinophil cytoplasm of the colon lamina propria of the Muc2−/− mice mouse. Arrows show mitochondria with a sparse matrix (B). Mast cell cytoplasm of the colon lamina propria of the Muc2−/− mice mouse. The arrow shows a mitochondrion with membrane disruption (C). Plasmocyte cytoplasm of the colon lamina propria of the Muc2−/− mice mouse. The arrows show «empties» and «hernies» in mitochondria
