# Supplementary material for: Changes in the Phenotype and Metabolism of Peritoneal Macrophages in Mucin-2 Knockout Mice and Partial Restoration of Their Functions In Vitro After L-Fucose Treatment
Source: Int J Mol Sci. 2024 Dec 24;26(1):13. doi: 10.3390/ijms26010013 (PMC11719744; doi:10.3390/ijms26010013)
Supplement: Supplementary file 1 [file ijms-26-00013-s001.zip › fig_S2.pdf]

Supplementary figure S2

A

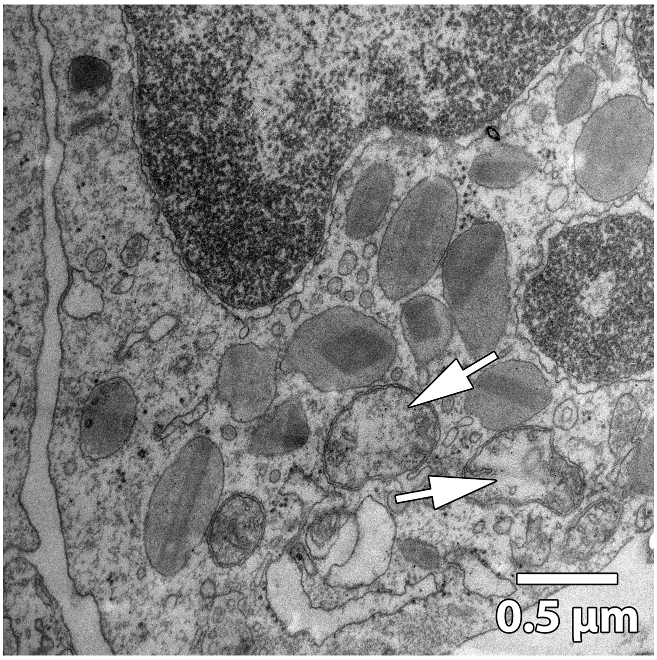

Eosinophil cytoplasm of the colon lamina propria of the Muc2-/- mouse. Arrows show mitochondria with a sparse matrix

B

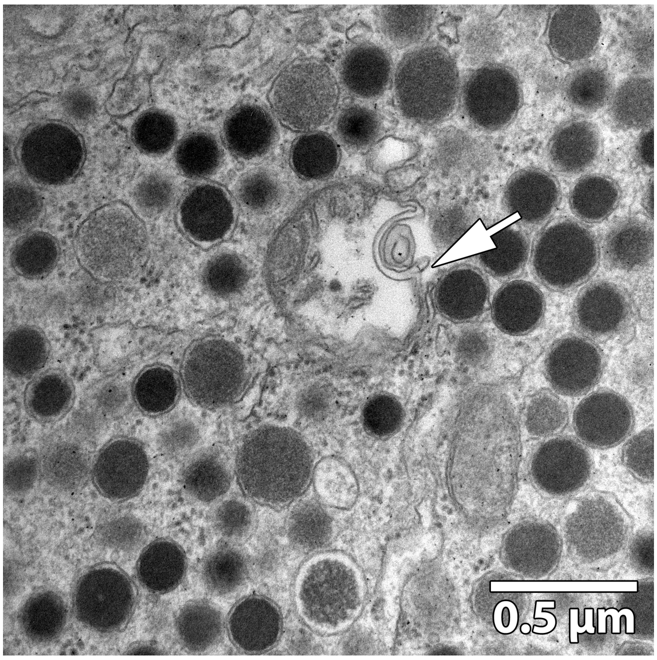

Mast cell cytoplasm of the colon lamina propria of the Muc2-/- mouse. The arrow shows a mitochondrion with membrane disruption

C

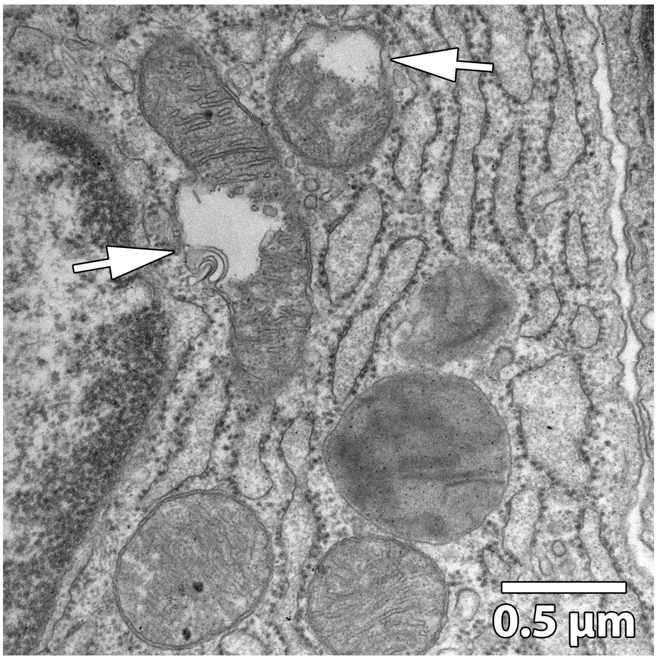

Plasmocyte cytoplasm of the colon lamina propria of the Muc2-/- mouse. The arrow shows «empties» and «hernies» in mitochondria
