# Supplementary figures and images for: Changes in the Phenotype and Metabolism of Peritoneal Macrophages in Mucin-2 Knockout Mice and Partial Restoration of Their Functions In Vitro After L-Fucose Treatment
Source: Int J Mol Sci. 2024 Dec 24;26(1):13. doi: 10.3390/ijms26010013 (PMC11719744; doi:10.3390/ijms26010013)

Supplementary figure S1

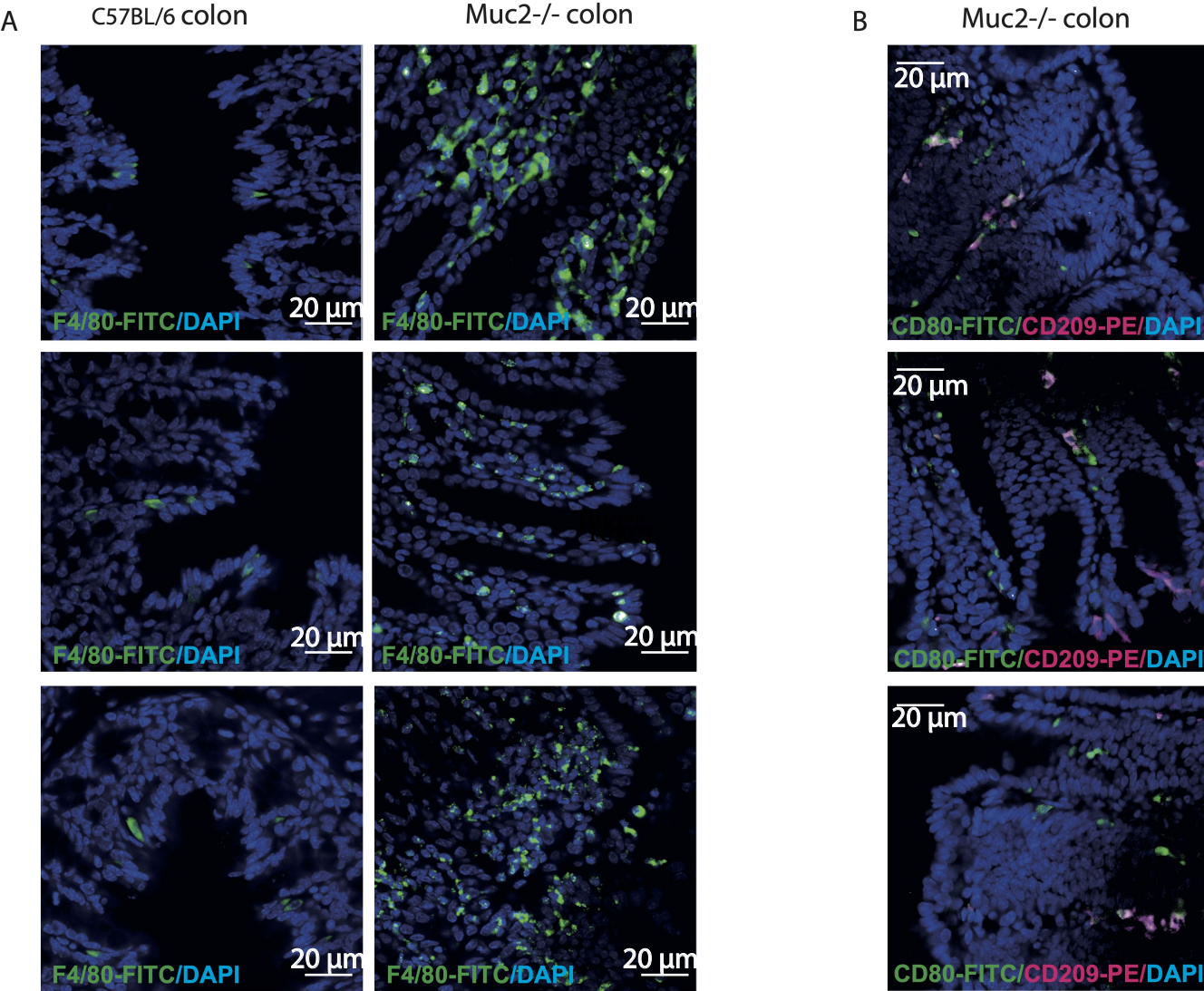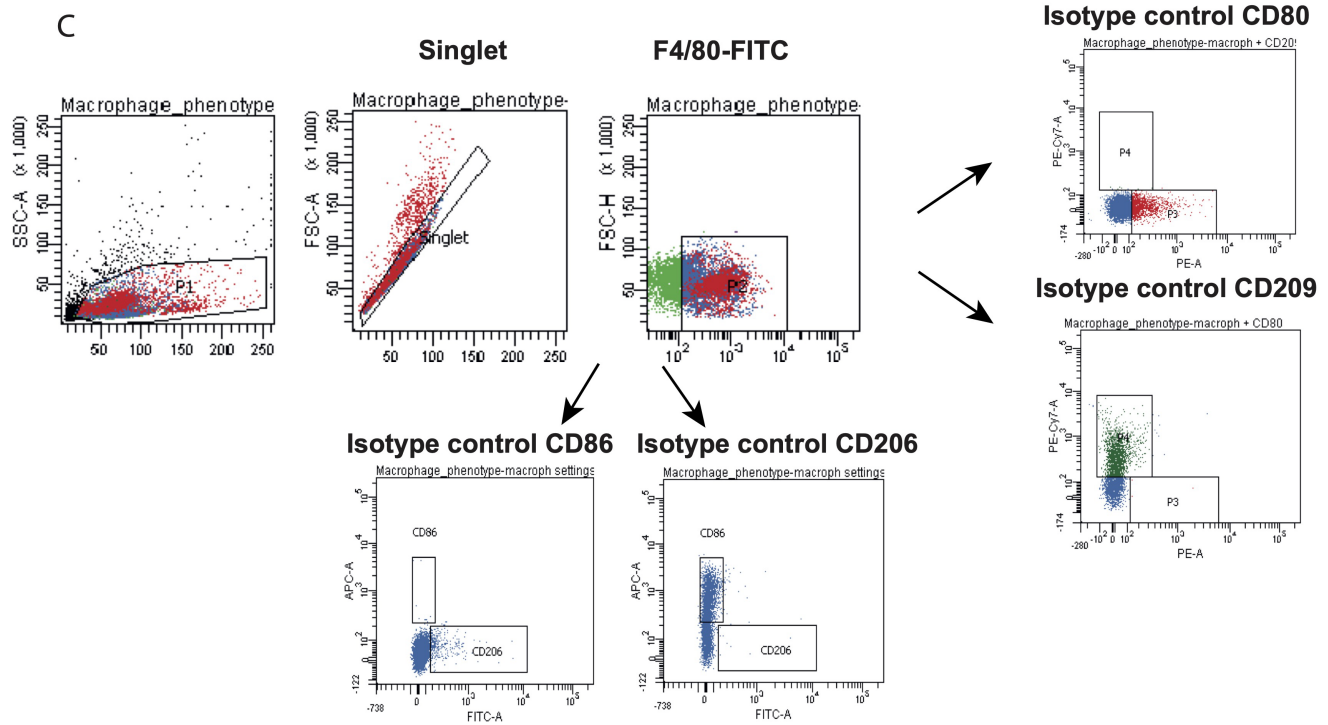

Supplement: Supplementary file 1 [file ijms-26-00013-s001.zip › fig_S1.pdf]
